# Protective effects of heme oxygenase-1 against severe acute pancreatitis via inhibition of tumor necrosis factor-α and augmentation of interleukin-10

**DOI:** 10.1186/s12876-017-0651-4

**Published:** 2017-08-24

**Authors:** Fei-hu Zhang, Yu-han Sun, Kai-liang Fan, Xiao-bin Dong, Ning Han, Hao Zhao, Li Kong

**Affiliations:** 1 Department of Emergency Center, Affiliated Hospital of Shandong University of Traditional Chinese Medicine, Jingshi Road No.16369, Jinan, Shandong Province 250011 China; 2 Department of Traditional Chinese Medicine, Jinan Municipal Organs Hospital, Jianguoxiaojingsan Road No.35, Jinan, Shandong Province 250001 China

**Keywords:** Heme oxygenase-1, Severe acute pancreatitis, Oxidative stress, Tumor necrosis factor-α, Interleukin-10

## Abstract

**Background:**

Heme oxygenase-1 (HO-1) is an inducible defense gene which plays a significant role in inflammation. HO-1 protects cells and tissues through the mechanism of anti-oxidation, maintaining microcirculation and anti-inflammation. The aim of the current study is to investigate the role of HO-1 on systemic inflammatory response in severe acute pancreatitis (SAP).

**Methods:**

Forty male Sprague-Dawley (SD) rats were randomly assigned into four groups: control group (*n* = 10); SAP group (*n* = 10), SAP model was induced by retrograde injection of 3% sodium taurocholate through pancreatic duct; HO-1 stimulation group (*n* = 10), SD rats were injected 75 μg/kg hemin intraperitoneally 30 min after induction of SAP; HO-1 inhibition group (*n* = 10), SD rats were injected 20 μg/kg Zinc porphyrin (Zn-PP) intraperitoneally 30 min after induction of SAP. After 24 h of SAP establishment, tissues were collected for HO-1, tumor necrosis factor-α (TNF-α) and interleukin-10 (IL-10) mRNA expression, and blood samples were collected for cytokines and biochemical measurements. Meanwhile, the histopathological changes of pancreas and liver tissues were observed.

**Results:**

The expression of HO-1 mRNA and protein were significantly induced by SAP in rat pancreas and liver. Hemin treatment significantly decreased oxidative stress and TNF-α in plasma and tissues, while the IL-10 was significantly increased. Pancreas and liver injury induced by SAP was markedly attenuated by Hemin treatment. Moreover, inhibition of HO-1 expression by Zn-PP administration aggravated the injury caused by SAP.

**Conclusions:**

Induction of HO-1 in early SAP may modulate systemic inflammatory response and prevent pancreas and nearby organs such as liver injury through inhibition of TNF-α and augmentation of IL-10.

## Background

Acute pancreatitis (AP), with a reported annual incidence of 13 ~ 45 cases per 100,000 people [1], and the mortality is up to 30% in severe cases [2], is one of the most common gastrointestinal disorders. It is widely accepted that inflammation plays a pivotal role in the pathogenesis of severe acute pancreatitis (SAP). The early acinar cell injury in SAP causes local inflammation, which subsequently activates the immune system inappropriately and eventually results in multiple organs dysfunction syndrome (MODS) [3]. Thus, the therapy strategy targeting to inhibit the pro-inflammatory cytokines and boost the anti-inflammatory cytokines attached much attention and might be an effective way for the treatment of SAP [4].

Several studies have demonstrated that heat shock proteins (HSPs) can inhibit both intrinsic and extrinsic pathways of apoptosis at multiple sites [5]. HSPs, which express in a variety of cells against stress and injury inciting stimulis, belong to a family of proteins which are highly conserved. Recent studies indicated that some HSPs, such as HSP-32/heme oxygenase-1 (HO-1), play important roles in the pathogenesis of SAP and some other several immune-mediated inflammatory diseases [6]. HO-1 (also referred to as HSP-32), an inducible isoform of heme oxygenase, catalyzes the degradation of heme into carbon monoxide (CO), iron and biliverdin [7]. Iron is sequestered by ferritin, and biliverdin is subsequently converted to bilirubin. Because of the anti-oxidization, anti-apoptotic, anti-proliferative, and anti-inflammatory effects of heme metabolites, HO-1 has been emerged as an important cytoprotective enzyme. Some studies showed that both transgenic overexpression and pharmacological activation of HO-1 alleviated and eventually eliminated the oxidative cell damage that occurs in some disease states [8]. Also, HO-1 plays an important role in mediating the pro-inflammatory effect of TNF-α and the anti-inflammatory effect of IL-10 [9, 10]. However, the role of HO-1 in the exocrine pancreas and its potential modulation role in pancreatic injury are still not fully elucidated [11].

In this study, we evaluated the effect of HO-1 on systemic inflammatory mediators: TNF-α and IL-10. Further research on the protective effects of HO-1 against SAP is necessary because it may be useful to improve organs function and survival rate via genetic or pharmacological strategies in SAP.

## Methods

### Animal ethics statement and experimental protocol

All animal experiments were conducted in accordance with the guidelines of the Shandong Committee on Animal Care of China which approved the study protocol. Male Sprague-Dawley (SD) rats weighting 220 g to 260 g were purchased from the Shandong Experimental Animal Center of Chinese Academy Science. All rats were housed in a temperature controlled (25 ± 1 °C) room under a 12-h light/12-h dark cycle with free access to drinking water and chow diet. Forty healthy male SD rats were randomly assigned into four groups: control group; SAP group; HO-1 stimulation group, Hemin (75 μg/kg; Sigma Chemical, St. Louis, MO) [12] was injected intraperitoneally 30 min after induction of SAP; and HO-1 inhibition group, Zn-PP (20 μg/kg; Sigma Chemical, St. Louis, MO) [9] was injected intraperitoneally 30 min after induction of SAP. Rats were anesthetized with sodium pentobarbital (40 mg/kg, intraperitoneally) and sacrificed 24 h after SAP establishment. Blood samples collected from the celiac artery were centrifuged and the serum were stored at −80 °C for the analysis of Amylase, Lipase, Alanine aminotransferase (ALT), Aspartate aminotransferase (AST), HO-1, TNF-α and IL-10 level. Pancreas and liver were immediately dissected from their attachments and divided for total RNA extraction. Portions of pancreas and liver were fixed in 40 g/L buffered formaldehyde for histological test.

### Establishment of SAP model

Rats were anesthetized by intraperitoneal injection with sodium pentobarbital (40 mg/kg; Sigma Chemical, St. Louis, MO). Rats were then retrogradely injected 3% sodium taurocholate (0.1 mL/100 g; Sigma Chemical, St. Louis, MO) through pancreatic duct and the pressure was maintained for 5 min [3].

### Measurement of HO-1, TNF-α and IL-10

The serum levels of HO-1, TNF-α and IL-10 were determined using enzyme-linked immunosorbent assay (ELISA) kits (EIAab, Shanghai, China) [3]. The mRNA levels of HO-1, TNF-α and IL-10 in tissues were determined using real-time PCR as described before and determined by the data from the real-time PCR instrument (ABI7900, Applied Biosystems, Foster City, CA) [3]. Briefly, total RNA was extracted from tissues with TRIzol reagent following the manufacturer’s instructions and aliquots of 5 μg of total RNA were reverse-transcribed using the first-strand cDNA synthesis kit (Promega A3500, Madison, WI) [3]. The cDNA was then amplified by polymerase chain reaction using specific primers for HO-1, TNF-α and IL-10, and β-actin was used as internal control [3]. The primers used were in Table 1. PCR reactions were performed under the following conditions: denaturation at 95 °C for 15 s, annealing at 60 °C for 20 s and extension at 72 °C for 30 s [3].

**Table 1:** PCR primer sequences (5′-3′)

Gene	Forward primer	Reverse primer
HO-1	ACCCCACCAAGTTCAAACAG	GAGCAGGAAGGCGGTCTTAG
TNF-α	CCCAATCTGTGTCCTTCTAACT	CACTACTTCAGCGTCTCGTGT
IL-10	GGCTCAGCACTGCTATGTTGCC	AGCATGTGGGTCTGGCTGACTG
β-actin	TGGTGGGTATGGGTCAGAAG	GACAATGCCGTGTTCAATGG

### Serum biochemical assays

The serum levels of Amylase, Lipase, ALT and AST were measured using automatic biochemical analyzer (UniCel DxC800, Beckman Coulter, CA) following the instructions.

### Histopathological analysis

Paraffin-embedded pancreas and liver were cut into 5 μm thick sections, and stained with hematoxylin and eosin for light microscopic examination as described before [3]. Histological assessment was performed by an investigator blind to group assignment, and the pathological scores of pancreas and liver samples were determined by the standard of Schmidt et al. [13] and Sass et al. [14].

### Statistical analysis

Data were analyzed using SPSS 16.0 software. All data in text and figures were expressed as mean ± SEM, and results were compared using the one-way analysis of variance followed by Tukey’s test and unpaired Student’s *t* test. A *p*<0.05 was considered to be statistically significant.

## Results

### Differential expression patterns of HO-1, TNF-α and IL-10 in serum, pancreas and liver

**Fig. 1:**
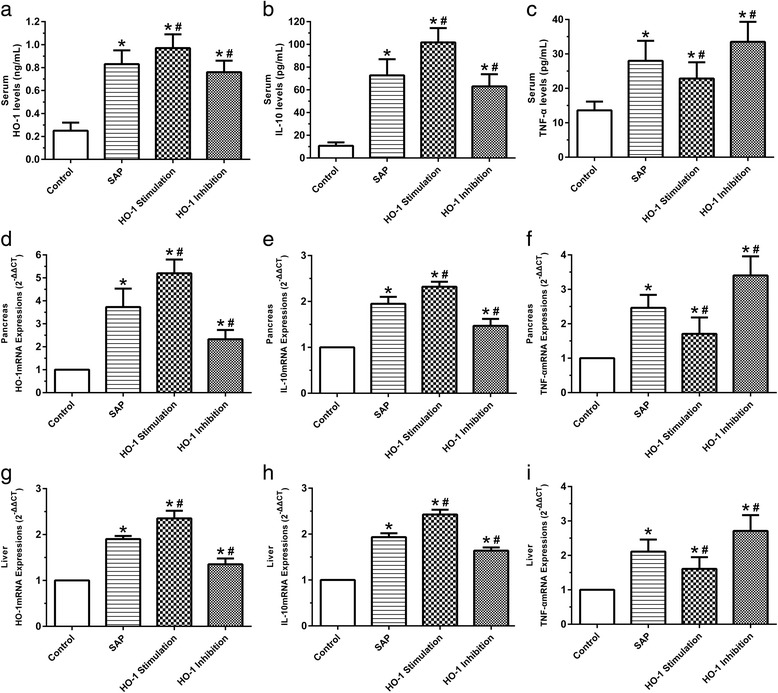
Differential expression patterns of HO-1, IL-10 and TNF-α in serum, pancreas and liver after 24 h of SAP surgery. **a,** HO-1 levels in serum; **b,** IL-10 levels in serum; **c,** TNF-α levels in serum; **d,** HO-1mRNA expressions in pancreas; **e,** IL-10mRNA expressions in pancreas; **f,** TNF-αmRNA expressions in pancreas; **g,** HO-1mRNA expressions in liver; **h,** IL-10mRNA expressions in liver; **i,** TNF-αmRNA expressions in liver. Data are presented as mean ± SEM (*n* = 10). ^*^ *p* < 0.05, compared with the control group; ^#^ *p* < 0.05, compared with the SAP group

Compared with the control rats, the HO-1, TNF-α and IL-10 levels in serum and also the mRNA levels in pancreas and liver were significantly increased by SAP after 24 h of surgery (*p*<0.05) (Fig. 1a–i). While hemin administration significantly increased HO-1 and IL-10 levels both in serum and in pancreas and liver (*p*<0.05) (Fig. 1a–h). Though hemin administration increased TNF-α in serum and its mRNA expressions in pancreas and liver (*p*<0.05) (Fig. 1c–i), it significantly decreased TNF-α induced by SAP after 24 h of surgery (*p*<0.05) (Fig. 1c–i). In addition, Zn-PP treatment increased HO-1 and IL-10 both in serum and in pancreas and liver (*p*<0.05) (Fig. 1a–h). However, Zn-PP treatment significantly decreased HO-1 and IL-10 level in serum, pancreas and liver induced by SAP (*p*<0.05) (Fig. 1a–h). Moreover, Zn-PP administration increased TNF-α in the serum and the expressions of TNF-αmRNA in the pancreas and liver (*p*<0.05) (Fig. 1c–i).

### Levels of biochemical parameters in serum

**Fig. 2:**
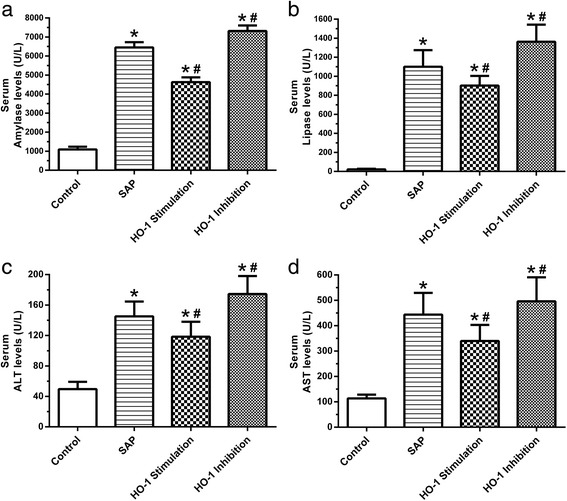
Levels of Amylase, Lipase, ALT and AST in serum after 24 h of SAP surgery. **a,** Amylase levels in serum; **b,** Lipase levels in serum; **c,** ALT levels in serum; **d,** AST levels in serum. Data are presented as mean ± SEM (*n* = 10). ^*^ *p* < 0.05, compared with the control group; ^#^ *p* < 0.05, compared with the SAP group

The levels of Amylase, Lipase, ALT and AST in the serum were significantly induced by SAP after 24 h of surgery (*p*<0.05) (Fig. 2a–d). Although hemin treatment increased the Amylase, Lipase, ALT and AST in the serum (*p*<0.05) (Fig. 2a–d), it significantly decreased these markers induced by SAP (*p*<0.05) (Fig. 2a–d). On the other hand, Zn-PP treatment significantly increased the level of Amylase, Lipase, ALT and AST in the serum (*p*<0.05) (Fig. 2a–d).

### Histopathological evaluation and scores of pancreas and livers

**Fig. 3:**
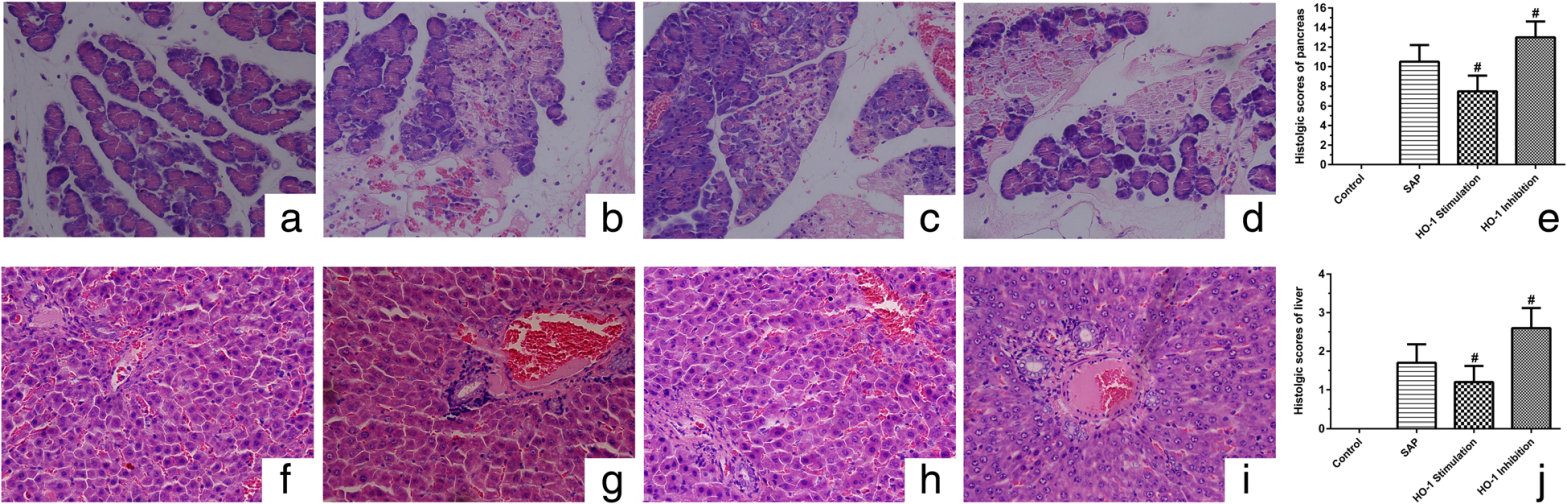
Histopathological evaluation of pancreas and livers after 24 h of SAP surgery (HE × 400). **a**, pancreas of control group; **b**, pancreas of SAP group; **c**, pancreas of HO-1 stimulation group; **d**, pancreas of HO-1 inhibition group; **e,** pathological scores of pancreas; **f**, liver of control group; **g**, liver of SAP group; **h**, liver of HO-1 stimulation group; **i**, liver of HO-1 inhibition group; **j,** pathological scores of liver. Pathological scores are presented as mean ± SEM (*n* = 10). ^#^ *p* < 0.05, compared with the SAP group

The structure of pancreas of control rats showed morphologically normal, while the pancreas of SAP rats displayed partly hemorrhage, necrosis and infiltration of neutrophile granulocyte. Heme admistraton relieved pathological damage in pancreas caused by SAP, including the integrity of pancreatic duct and less infiltration of neutrophile granulocyte, while Zn-PP treatment caused more severe pathological pancreas damages including large scale pancreatic and vascular necrosis as well as mass infiltration of neutrophile granulocyte (Fig. 3a–d). The pathological scores were significantly reduced by stimulation of HO-1, whereas enhanced by inhibition of HO-1(*p*<0.05) (Fig. 3e).

The hepatic cells in control rats, showing morphologically normal, were observed in cord-like arrangement, and the structure of hepatic lobe was clear. While the cytoplasm became loosened, and the Kupffer cell proliferated in hepatic sinusoid in SAP rats. There were less Kupffer cells in sinusoid and the morphology of the hepatic cells was normal after heme admistration. In addition, the hepatocytes showed spotty necrosis with more loosened cytoplasm and lymphocyte infiltration after Zn-PP treatment (Fig. 3f–i). HO-1 stimulation significantly reduced the pathological scores induced by SAP, while HO-1 inhibition by Zn-PP significantly enhanced the pathological scores. (*p*<0.05) (Fig. 3j).

## Discussion

Acute pancreatitis (AP), with severe complications and high mortality under severe condition which called SAP, is an inflammatory condition of the pancreas. A manifestation of the inflammatory response is a hallmark of AP. In early SAP, the acinar cell injury causes the pancreatic cells secret inflammatory mediators like TNF-α and IL-10, which extend the inflammatory response and cause the organ injury. Our study showed that in the early stage of SAP, the HO-1 gene expression increased in the pancreas and liver. Also, induction of HO-1 by hemin treatment significantly increased plasma IL-10 and also decreased TNF-α, which modulated the inflammatory reaction, oxidative damage, and organs injury. These results demonstrated the beneficial effects of HO-1 in early SAP through mediating the systemic inflammatory response, indicating that HO-1 plays an important role in protecting pancreas and nearby organs from injury under SAP [3, 11, 15–17].

SAP is associated with the induction of several cytokines, including pro-inflammatory and anti-inflammatory mediators [18–20]. Some studies have demonstrated that TNF-α, which is secreted by activated macrophage and lymphocyte, plays an important role in the occurrence and development of SAP [21]. Induction of TNF-α subsequently induces the expression and secretion of IL-6, IL-8 as well as itself, causing the inflammatory cascade and the uncontrolled releasing of inflammatory mediators [18], which eventually cause the organs failure or even death. In contrast, IL-10, which is produced by macrophages, Th2 cells, hepatocytes and stellate cells, has the anti-inflammation effect in inflammatory diseases [22]. IL-10 inhibits the synthesis of pro-inflammatory cytokines, such as IL-2, IL-3 and TNF-α, and also prevents MODS caused by SAP [20, 23]. In our study, induction of HO-1 by Hemin in early SAP significantly decreased TNF-α in plasma and tissues, while the plasma and tissues IL-10 level was increased. In contrast, inhibition of HO-1 expression by Zn-PP treatment increased TNF-α and decreased IL-10 in plasma and tissues. So, it suggested that HO-1 plays a protective role in SAP through anti-inflammatory pathways. The heme metabolites catalyzed by HO-1 have anti-inflammatory effects through induction of IL-10 [10, 24]. It is still need to illuminate whether the protective effects of HO-1 in SAP is attributed to its metabolites, CO or the antioxidant bilirubin [25–28]. Dependent on the modulation of p38 mitogen-activated protein kinase (MAPK), CO showed anti-inflammation effect through inhibition of pro-inflammatory cytokines production [29, 30]. Our data demonstrated that the induction of HO-1 in early SAP can inhibit the inflammatory response through mediating the cytokines production and mitigate the damage to pancreas and nearby organs such as liver, indicating that HO-1 may function as therapeutic target for the treatment of SAP.

HO-1 is a stress-inducible enzyme which catalyzes the degradation of heme into CO, iron and biliverdin [7]. Under oxidative stress, such as inflammation and ischemia-reperfusion, HO-1 is induced and protects organs from damage, which in part by the anti-inflammatory effect of heme metabolites [31–35]. The expression of genes responsible for oxidative stress, especially HO-1 [16, 36, 37], are remarkably upregulated in the course of SAP, which suggests the existence of a compensatory mechanism against stress. Like most of the antioxidants, which protect organs from oxidative stress caused apoptosis and failure [38–40], hemin treatment induced HO-1 expression in early SAP and mitigated pancreas injury caused by oxidative stress and inflammation. In contrast, inhibition of HO-1 expression by Zn-PP aggravated the organs injury in SAP. These results indicated that induction of HO-1 in SAP may provide a new and effective therapeutic strategy for SAP.

## Conclusions

In summary, our study demonstrated that HO-1 induction mitigated the pancreas injury through decreasing oxidative stress and TNF-α production, and also increasing IL-10 production in SAP. HO-1 overexpression also decreased the markers associated with pancreas and liver injury. Induction of HO-1 in early SAP, which reduced systemic inflammatory response and organs injury, may provide a new and effective therapeutic treatment for SAP.

## Abbreviations

ALT: Alanine aminotransferase
AP: acute pancreatitis
AST: Aspartate aminotransferase
CO: carbon monoxide
ELISA: enzyme-linked immunosorbent assay
HO: Heme oxygenase
HSPs: heat shock proteins
IL: Interleukin
MAPK: mitogen-activated protein kinase
MODS: multiple organs dysfunction syndrome
SAP: severe acute pancreatitis
SD: Sprague-Dawley
TNF: Tumor necrosis factor
Zn-PP: Zinc porphyrin
